# Synthesis and Biological Activity of Antimicrobial Agents, 2nd Volume

**DOI:** 10.3390/antibiotics12111564

**Published:** 2023-10-26

**Authors:** Maria Fernanda N. N. Carvalho

**Affiliations:** Centro de Química Estrutural, Institute of Molecular Sciences and Departamento de Engenharia Química, Instituto Superior Técnico, Universidade de Lisboa, Avenida António José de Almeida, 12, 1000-043 Lisboa, Portugal; fcarvalho@tecnico.ulisboa.pt

## Actual Antimicrobial Needs and Tentative Solutions

Microorganisms are abundant and necessary. In nature, many types of microorganisms exist, some of which are essential for human and environmental life. However, in the wrong places and/or at inadequate concentrations, microorganisms can cause infections and become a threat to human life and/or the environment. According to the European Centre for Disease Control, 90,000 fatalities *per* year are estimated to be caused by infections acquired in hospitals [1].

Antibiotics, of which penicillin, discovered by Alexander Fleming [2], was the first commercially available, were successfully used for many years to fight infections. However, the misuse and abuse of antibiotics ended up with microorganisms’ adaption and resistance to an increasing number of them. Consequently, antibiotic microbial resistance (AMR) is nowadays a serious obstacle to defeating an increasing number of infections. The World Health Organization (WHO) nominated in 2017 three priority levels of awareness concerning 12 families of bacteria that include *Pseudomonas aeruginosa* (Priority 1: Critical), *Staphylococcus aureus* (Priority 2: High), and *Streptococcus pneumoniae* (Priority 3: Medium), for which WHO claimed new antibiotics are urgently necessary [3].

However, not just bacteria cause risks to human life. Infections by fungi [4] and viruses are also a subject of high concern. In hospitals, infection by *Candida auris* is currently a matter of preoccupation [5], while the SARS-CoV-2 virus and its variants continue to cause fatalities [6,7]. A variety of approaches are under way that encompass conventional and less conventional methodologies [8] to try to face the threats caused by bacteria, fungi, and viruses.

In this Special Issue of *Antibiotics*, the *focus* on the strategies to overcome infections is made on the *Synthesis and Biological Activity of Antimicrobial Agents*.

The search for new organic or inorganic molecules or nanomaterials with potential applications as antimicrobials follows two main routes. The traditional strategy is based on the synthesis/characterization and experimental evaluation of the antimicrobial properties of compounds, followed by the study of their mechanisms of action. The emergent, in silico, strategy is based on computer modeling to design potentially active molecules, molecular docking, and dynamic simulation to obtain insights into the interactions involved, among other computational approaches. An in silico strategy can be used to direct the laboratorial synthesis and evaluation of the antimicrobial in vitro or in vivo activities of selected molecules.

A vast pool of new antimicrobials is necessary to find a few that meet the requirements for pharmaceutical applications. In 2017, out of 51 electable active antimicrobials, just 8 fulfilled the WHO requisites [9] and proceeded to Phase III clinical evaluation. From 2017 to 2020, the new antibiotics approved include eravacycline, delafloxacin, cefiderocol, plazomicin, and one β-lactamase inhibitor [10].

To fulfill all the steps necessary to attain and surpass Phase III of the clinical trials, a considerable investment is necessary.

In 2019–2020, a sudden boost of the SARS-CoV-2 virus forced investment in the search for new antimicrobials and strategies to face the dramatic consequences of the COVID-19 pandemic. The use of existing drugs [contribution 1] was the first approach to fighting the virus, while several types of vaccines (messenger RNA, protein subunit, viral vector, whole virus) were under development. Vaccines are a valuable preventive strategy to sustain virus proliferation and decrease morbidity and mortality. However, microorganisms adapt and develop new pathogenic strains that spread. That is the case of the new SARS-CoV-2 BA.2.86 variant (August 2023) whose consequences and resistance to existing vaccines are under evaluation.

Although vaccines are an essential preventive tool, they cannot combat acute infections caused by pathogenic microorganisms. Therefore, in addition to adapted vaccines that act on new variants of bacteria (e.g., *Koch’s bacillus* that causes tuberculosis) or viruses (e.g., influenza) and prevent their proliferation, it is necessary to pursue efforts to find alternatives to face microbial resistance to the antibiotics under use. To do so, laboratory synthetic and computational methodologies are under way, some of which can be found in this Special Issue of *Antibiotics*: *Synthesis and Biological Activity of Antimicrobial Agents, 2nd volume*.

The synthetic approaches involve: (i) the design, synthesis, and biological activity evaluation of new molecules; (ii) the structural optimization of molecules that are biologically significant through changes, such as, for example, the position of the substituents at aromatic rings as mentioned in contribution 2 or the use of different halides [contribution 3]. Such apparently slight modifications can modify and eventually switch on/off the activity of the molecules.

Currently, combined computational and synthetic strategies are more and more common. Examples in this Special Issue are the in silico approach to the design of fusidic acid derivatives to fight Gram-positive *Staphylococcus* type bacteria and skin infections [contribution 4] or docking methodologies to obtain insights into the interactions of selected enzyme inhibitors with the Zn^2+^ ion of β-latamase [contribution 5]. Enzyme inhibitors, able to deactivate the metal site of enzymes (expressed by the microorganisms), are often responsible for inhibiting the activity of the antimicrobials. Molecules such as pyrrolyl benzohydrazides were shown in [contribution 6] to be active on the inhibition of enoyl ACP reductase and DHFR enzymes, thus reducing the *M. tuberculosis* proliferation.

A step forward featuring the therapeutic use of molecules for targeting enzymes is their combination with existing antibiotics. That is the case of β-Lactam-Metallo-β-Lactamase inhibitors (e.g., BP2), which remove zinc from the β-Lactamase active site and preclude meropenem antibiotic loss of activity [contribution 5].

Synergic effects between several bioinspired synthetic peptides and itraconazole (ITR) were found to highly potentiate the azole activity against *C. neoformans* fungi responsible for meningitis while decreasing its toxicity [contribution 7]. The process involves pore formation, which facilitates ITR moving through the cell membrane of the fungus. Concomitant overproduction of ROS promoted by combined ITR/peptides further enhances the antifungal activity. The related Mo-CBP_3_-PepI peptide was found to be efficient against *Klebsiella pneumoniae* bacteria, which is responsible for severe infections in patients with immune-depressed systems. The *modus operandi* in fungi and bacteria is analogous, as far as reported. These peptides act at the microbial cell membrane through hole formation and ROS overproduction, thus controlling bacterial proliferation and film formation [contribution 8]. Other compounds, such as chalcones or flavones mentioned in [contribution 9], act through distinct mechanisms, e.g., disruption of the membrane with leakage of the cell content.

Synthesis and screening of the antimicrobial properties of organic molecules created a pool of compounds with antimicrobial properties, some of which are referred to in the Special Issue as, for example, sulfenimines [contribution 10], pyrrolyl benzohydrazides [contribution 6], chalcones, flavones, flavanones, quinolinequinones, [contribution 3] thiourea derivatives (thiazole, benzothiazole, pyridine, and pyrimidine) [contribution 2], and peptides [contributions 5 and 7], some of which have promising activity towards clinically resistant bacterial strains.

In contrast to the large number of organic molecules, a scarce number of biologically active coordination compounds have been reported. However, the presence of the metal can tune quite distinct biological mechanisms and types of biological activities. That is the case of camphorimine complexes, which, depending on the metal, can display high antimicrobial [contribution 11] and/or anticancer activity [11].

Design, synthesis, activity assessment, and comprehension of the mechanisms of antimicrobial action are essential steps in a molecule´s use as therapeutic agents or as disinfectants. However, toxicity is often a drawback concerning therapy, while integrity and action time are important parameters for disinfection processes.

In parallel to organic molecules or coordination compounds, nanomaterials have been developed as antimicrobials. Nanomaterials with photo-induced activity are particularly attractive since they use the electric effect triggered by sunlight energy to kill bacteria and molecular oxygen to overproduce ROS. In addition, photo-antibacterials have low potential to promote microbial resistance. In this Special Issue, the photo-antibacterial activity of two-dimensional nanomaterials (2D-NMs) is reviewed in [contribution 12], showing that they are especially active against Gram-positive bacteria. The parameters that drive the photo-antibacterial activity, the role of oxygen, and the beneficial effect of metal dopants are discussed. The dopant (metal or non-metal) acts on the electronic properties of the nanomaterial by narrowing the band gap, lowering the recombination rate, and enhancing the photogenerated current, thus increasing the activity of the 2D-NMs. The specific biological properties of the dopant, which include a variety of transition metals, fine-tune the target microorganisms. Composites obtained from 2D-NMs sometimes require stabilization (e.g., poly-thiophenes, glycol-chitosan, etc.), forming hybrid materials that preclude leach-out of the metal. Biocompatibility can be enhanced by incorporating polymers or other materials into the 2D-NM composites. A focus on the time of action of the photo-antimicrobials is made in [contribution 12] regarding applications such as water disinfection.

Toothpastes for oral care are one of the fields of application of antimicrobials as disinfectants. In toothpastes, fluoride is used to reduce the growth of cariogenic microorganisms. However, excess fluoride may be disadvantageous, especially for children, since its ingestion may lead to dental fluorosis. In this Special Issue, three papers are devoted to the study of the effects of reducing the amount of fluoride with the concomitant addition of hexametaphosphate nanoparticles (HMPnano) [contribution 13], polyols/sodium trimetaphosphate (TMP) [contribution 14], or calcium glycerophosphate (GaGP) [contribution 15]. Each of the above-mentioned works reports successful results concerning the cariogenic effect of their approach on the biofilms of *S. mutans* and *C. albicans*, even in the absence of fluoride.
